# ^18^F-FDG PET/MR reveals specific brain metabolic features in Parkinson’s disease with frailty

**DOI:** 10.3389/fnagi.2025.1624203

**Published:** 2026-01-22

**Authors:** Guoyang Li, Wenli Zhang, Fengju Mao, Hong Zhao, Long Zhao, Yang Yang, Chang Sun, Lu Liu, Xiangcheng Wang, Xiaoguang Luo

**Affiliations:** 1 Department of Neurology, Second Clinical Medical College of Jinan University, Shenzhen People’s Hospital, Guangdong, China; 2 Shenzhen Clinical Research Centre for Geriatrics, Shenzhen People’s Hospital, Shenzhen, Guangdong, China; 3 Department of Nuclear Medicine, Shenzhen People’s Hospital (The Second Clinical Medical College, Jinan University, The First Affiliated Hospital, Southern University of Science and Technology), Shenzhen, Guangdong, China; 4 United Imaging Healthcare Group Co., Ltd., Shanghai, China

**Keywords:** ^18^F-FDG PET/MR, Frailty, MoCA, Parkinson’s disease, UPDRS-III

## Abstract

**Background:**

Frailty is significantly more prevalent in individuals with Parkinson’s disease (PD) than in general population, yet the underlying neuropathophysiological mechanisms remain poorly understood. This study aimed to characterize the clinical features and cerebral metabolic patterns of frail PD patients using [^18^F]-fluorodeoxyglucose positron emission tomography (^18^F-FDG PET), and to explore the potential pathophysiological mechanisms.

**Methods:**

A total of 64 PD patients treated at Shenzhen People’s Hospital underwent ^18^F-FDG PET/MR imaging during June-December 2024. Age- and sex-matched healthy controls were also recruited (*n* = 17). For PD patients, frailty was assessed using the Fried criteria. Patient demography, cognitive performance, and clinical variables—including UPDRS-III scores—were compared between frail and non-frail PD patients. Regional brain metabolism, measured as ^18^F-FDG SUVr, was analyzed in brain regions defined by the Automated Anatomical Labeling (AAL) atlas.

**Results:**

Among the PD cohort (mean age: 66.86 ± 6.97 years; 30 female), 34 were classified as non-frail (mean age: 64.29 ± 6.61 years; 16 female) and 30 as frail (mean age: 69.77 ± 6.17 years; 14 female). Compared to the non-frail group, frail PD patients were significantly older (*P* = 0.001) and exhibited more severe motor symptoms (UPDRS-III, *P* < 0.001; modified Hoehn & Yahr (Modified H&Y) stage, *P* = 0.028), along with greater cognitive impairment (*P* < 0.001). Although the daily levodopa equivalent dose did not differ significantly between groups (*P* = 0.076), a trend toward higher dosage was observed in the frail group. ^18^F-FDG PET/MR analysis revealed significantly reduced glucose metabolism in 13 brain regions in frail PD patients compared to non-frail patients: Frontal_Mid_L (*P* = 0.0056), Frontal_Mid_Orb_L (*P* = 0.0045), Frontal_Inf_Tri_R (*P* = 0.0053), Occipital_Mid_L (*P* = 0.0035), Occipital_Inf_L (*P* = 0.0053), Parietal_Inf_R (*P* = 0.0003), Angular_L (*P* = 0.0015), Angular_R (*P* = 0.0003), Caudate_L (*P* = 0.0052), Caudate_R (*P* = 0.0019), Temporal_Mid_R (*P* = 0.0040), Temporal_Inf_L (*P* = 0.0048), and Temporal_Inf_R (*P* = 0.0046). Correlation analyses revealed distinct region-function associations in the cognitive domains of frail PD patients. Regression analysis indicated that hypometabolism in the Temporal_Inf_R was significantly associated with UPDRS-III scores in the frail group.

**Conclusion:**

Frailty in PD is associated with advanced age, greater motor severity, and possibly increased medication needs. Frail PD patients exhibit specific patterns of cerebral hypometabolism and more severe cognitive deficits. Distinct brain regions are differentially associated with specific cognitive domains. Notably, reduced metabolism in the Temporal_Inf_R is significantly related to motor symptom severity in frail PD, suggesting a key region in the pathophysiology of frailty in PD.

## 1 Introduction

Parkinson’s disease (PD) is a progressive neurodegenerative disorder and the common neurodegenerative disease among the elderly. It is characterized by motor symptoms—such as tremor, rigidity, bradykinesia, and postural instability—as well as a range of non-motor symptoms, including sleep disturbances, depression and so on. The primary pathological hallmark of PD is the degeneration of dopaminergic neurons in the substantia nigra pars compacta (SNpc) and subsequent dopamine depletion in the striatum and the formation of Lewy bodies within neurons (Ka, 2002; Kalia and Lang, 2015; Liu et al., 2025). In China, the prevalence of PD among adults aged ≥ 65 years is approximately 17 per 1,000 and increases with age, placing a considerable burden on families and society (Zhang et al., 2005).

Frailty is a clinical syndrome marked by diminished physiological reserves, increased vulnerability to stressors, and impaired capacity to maintain homeostasis. It manifests as a non-specific state of increased risk for adverse health outcomes even after minor stressors (Fried et al., 2001; Thillainadesan et al., 2020). Fried et al. defined frailty using five criteria: slow gait speed, reduced grip strength, low physical activity, fatigue, and unintentional weight loss. Frailty is highly prevalent in older adults and is associated with numerous negative outcomes, including falls, disability, cognitive impairment, dependency, and even mortality (Fried et al., 2001). It has also been shown to predict adverse events such as hospitalizations, acute and chronic illness, and functional decline (Makizako et al., 2015) and other adverse outcomes (Rothman et al., 2008). Physical frailty is linked to social withdrawal, elevated cardiovascular risk factors, and cognitive decline or depression—factors that can lead to chronic malnutrition, oxidative stress, and systemic inflammation, thereby compounding the health consequences of frailty (Clegg et al., 2013; Robertson et al., 2013; Karway et al., 2025). Given its broad health impact, successful interventions for frailty can benefit both individuals and reduce healthcare system burdens globally (Clegg et al., 2013). Importantly, the prevalence of frailty is markedly higher in PD patients (29%–67%) than in age-matched older adults without PD (approximately 10%) (Collard et al., 2012; Roland et al., 2012; Liotta et al., 2017; Renne and Gobbens, 2018).

Previous studies have begun to explore associations between frailty and brain structure. For example, frail individuals show reduced gray matter volume in regions such as the cerebellum, hippocampus, and medial frontal cortex (Chen et al., 2015). Larger cerebellar gray matter volumes are positively associated with faster gait speed and better cognitive processing, suggesting that both gait and cognition are closely linked to frailty (Nadkarni et al., 2014). Smaller prefrontal areas have been associated with slower information processing and reduced gait speed (Rosano et al., 2012). Notably, frailty and cognitive impairment are closely intertwined; executive function, attention, visuospatial skills, and memory—all domains affected in PD—are also closely linked to gait speed (Amboni et al., 2013; Lin et al., 2019). Notably, frailty assessments have been shown to predict future cognitive decline and dementia over several years (Raji et al., 2010; Aranda et al., 2011; Doba et al., 2012).

^18^F-fluorodeoxyglucose (FDG), a glucose analog, is a commonly used radiotracer in neuroimaging. Cerebral glucose metabolism reflects local neuronal integrity and synaptic activity.^18^F-FDG uptake increases with synaptic activity and decreases with neuronal dysfunction, and has become a crucial molecular imaging modality to quantify metabolic dysfunction in neurodegenerative diseases (Mergenthaler et al., 2013; Chen et al., 2025). In addition to PET, the integrated PET/MR system provides simultaneous PET and high-resolution MR acquisitions, substantially reducing multimodal registration errors and offering a reliable platform for investigating brain metabolism in frail patients with PD.

In this study, we aimed to investigate the metabolic brain patterns associated with frailty in PD using ^18^F-FDG-PET/MR. By identifying brain regions associated with frailty risk and clinical features, we hope to uncover potential neuroimaging biomarkers and better understand the neuropathophysiological basis of frailty in PD. Furthermore, such biomarkers could serve as intermediate targets for monitoring the effectiveness of preventive interventions.

Based on the above, this study focuses on identifying brain metabolic patterns in PD patients with frailty and exploring the associations between brain metabolism, clinical characteristics, and frailty. Specifically, we aimed to:

(1)    Characterize the brain metabolic patterns of PD with frailty;
(2)    Examine the associations between brain metabolism and cognitive and motor function in frail PD patients;
(3)    Explore the underlying neuropathophysiological mechanisms of frailty in PD.

## 2 Materials and methods

### 2.1 Study design

This was an observational, cross-sectional neuroimaging study focused on frailty in patients with Parkinson’s disease (PD). All participants, including patients and healthy controls, provided written informed consent prior to undergoing PET/MR scans. The study protocol was approved by the Ethics Committee of Shenzhen People’s Hospital.

### 2.2 Inclusion and exclusion criteria

All patients underwent comprehensive clinical and neurological evaluations, as well as neuroimaging assessments including simultaneous brain ^18^F-FDG PET/MR imaging.

Inclusion criteria: all patients met the 2015 Movement Disorder Society (MDS) Clinical Diagnostic Criteria for PD (Postuma et al., 2015).

Exclusion criteria were as follows:

(1)    Evidence of intracranial pathology (e.g., stroke, brain tumor, or traumatic brain injury) on MRI;
(2)    Comorbid conditions that could substantially affect study outcomes, including severe cardiovascular or pulmonary disease, diabetes mellitus, heavy smoking or alcohol consumption, active malignancies, or major depressive disorder;
(3)    Parkinsonian syndromes other than idiopathic PD;
(4)    Inability to undergo MRI or PET scans due to claustrophobia or other contraindications;
(5)    Hoehn & Yahr stage 4 PD;
(6)    Severe dementia or comorbid dementia of other etiologies (e.g., Alzheimer’s disease or dementia with Lewy bodies) that would interfere with scale-based assessments;
(7)    Refusal to participate in the study.

The control group included age- and sex-matched individuals with no history of neurological disorders (e.g., PD, Alzheimer’s disease, stroke, traumatic brain injury, brain tumors, or inflammatory CNS conditions) or other conditions potentially affecting the study (e.g., diabetes, depression, significant smoking or alcohol use history).

Clinical data collected for all participants included date of birth, sex, and education level. For PD patients, additional clinical information was collected, including disease duration, age of onset, levodopa equivalent daily dose (LEDD) (Tomlinson et al., 2010), and medical history. Parkinson’s disease (PD) severity was staged using the Hoehn and Yahr Staging Scale, a validated tool for assessing motor symptom progression in PD (Hoehn and Yahr, 2011). Neurological assessments included: Montreal Cognitive Assessment (MoCA) to evaluate cognitive function, covering executive function (5 points),attention (6 points),naming (3 points), abstraction (2 points),language (3 points),delayed recall (5 points),and orientation (6 points). Scores range from 0 to 30, with higher scores indicating better cognitive performance (Nasreddine et al., 2005). Motor function was assessed using the Hoehn and Yahr staging scale and the Unified Parkinson’s Disease Rating Scale Part III (UPDRS-III),with higher scores reflecting more severe motor symptoms (Goetz et al., 2008; Chialà et al., 2018).

### 2.3 Frailty assessment

Frailty phenotype was assessed based on the Fried criteria, the most widely used method in clinical research. The five components included: Slowness: defined as walking speed ≤ 0.8 m/s over a 6-m walk. To minimize the effects of PD motor fluctuations, assessments were performed during the “on” state—when dopaminergic treatment is effective and symptoms are controlled (Olanow et al., 2001); Weakness: measured with an electronic dynamometer, defined as ≤25 kg for men and ≤18 kg for women; Low physical activity: defined as <383 kcal/week for men and <270 kcal/week for women. Energy expenditure was estimated based on walking speed: 3.1 kcal/kg/h at 4 km/h, and 4.4 kcal/kg/h at 6 km/h; Exhaustion: defined as feeling that “everything is an effort” on ≥3 days in a week; Unintentional weight loss (Weight loss): defined as ≥5% body weight loss within 1 year without dieting, exercise, or surgical intervention; Participants meeting three or more criteria were classified as frail, while those with one or two criteria were classified as non-frail (Fried et al., 2001).

### 2.4 PET and MRI acquisition and analysis

#### 2.4.1 PET/MRI image acquisition

All PET/MR scans were performed at the Department of Nuclear Medicine, Shenzhen People’s Hospital. After intravenous injection of 370 MBq of ^18^F-FDG, participants underwent simultaneous PET/MRI scanning 60–90 min later. Foam padding was used to stabilize the head and minimize motion artifacts. MRI sequences included:T1-weighted (T1w): TE = 3 ms, TR = 7.8 ms, flip angle = 100°, voxel size = 0.67 × 0.67 × 0.67 mm^3^, FOV = 24 cm; BOLD fMRI: TE = 30 ms, TR = 2 s, flip angle = 90°, voxel size = 3.5 × 3.5 × 3.5 mm^3^, FOV = 22.4 cm, 240 repetitions. ^18^F-FDG PET data were acquired over 10–30 min (median: 27.4 min) and reconstructed using an ordered subset expectation maximization (OSEM) algorithm with a voxel size of 2.34 × 2.34 × 2 mm^3^.

#### 2.4.2 FDG PET/MR image analysis

Positron emission tomography/MR images were processed using Statistical Parametric Mapping software (SPM8^1^) to normalize them to the Montreal Neurological Institute (MNI) space. Rigid-body registration using normalized mutual information was applied to co-register PET images to the individual’s T1-weighted MRI. Spatial normalization and tissue segmentation were performed on the T1w image, which was then used to warp the co-registered PET image into MNI space. The Automated Anatomical Labeling (AAL) atlas was applied to extract regional PET data. This anatomically defined brain atlas includes 116 regions (90 cerebral and 26 cerebellar) based on structural and functional brain characteristics, and offers high spatial resolution and anatomical accuracy. Standardized uptake value ratios (SUVr) for each region were calculated using the cerebellum as the reference region. To explore differences in FDG uptake between groups, analysis of covariance (ANCOVA) was performed on each brain region with age and sex as covariates. Regions of interest (ROIs) were identified as brain regions with significant SUVr differences in ANCOVA analysis after false discovery rate (FDR) correction for multiple testing.

### 2.5 Statistical analysis

Statistical analyses were conducted using SPSS (version 27.0), R (version 4.2.3), and Python (version 3.11.4). Two-sample Student’s *t*-tests were used for continuous variables, and chi-square tests for categorical variables. The Shapiro-Wilk test was used to assess normality of distributions. For non-normally distributed variables, non-parametric tests were applied. For normally distributed continuous variables, analysis of variance (ANOVA) was used to compare differences among groups. ANOVA results for multiple brain areas were corrected for multiple testing using the Benjamini-Hochberg FDR correction method, with adjusted *p*-value < 0.05 as significant threshold. Regression analyses were conducted to identify brain regions associated with motor symptoms in patients without dementia. Spearman correlation coefficients were calculated to assess the associations between ROIs and cognitive scores. A *p*-value < 0.05 (two-tailed) was considered statistically significant.

## 3 Results

As shown in Table 1, the study included PD patients who attended the Parkinson’s Disease Specialty Clinic at Shenzhen People’s Hospital (Second Clinical Hospital of Jinan University and First Affiliated Hospital of Southern University of Science and Technology) between June 2024 and December 2024, along with a corresponding number of healthy controls. Demographic and clinical data for 64 PD patients and 17 healthy controls are presented in Table 1. A total of 81 subjects were included in this study, consisting of 64 PD patients and 17 healthy controls. PD patients [mean age 66.86 ± 6.97 years, 30 females (46.88%)] were compared with healthy controls [mean age 62.88 ± 5.52 years, 11 females (64.71%)]. Further analysis compared 34 non-frail PD patients (mean age 64.29 ± 6.61 years, 16 females (47.06%)] with 30 frail PD patients (mean age 69.77 ± 6.17 years, 14 females (46.67%)]. The results indicated no significant statistical differences between PD patients and healthy controls in terms of gender (*P* = 0.975), disease duration (*P* = 0.418), PD subtype (tremor-dominant,akinetic-rigid,mixed) (*P* = 0.297), and education level (*P* = 0.709). Comparing non-frail and frail PD patients, significant differences were observed in age (*P* = 0.001), disease severity (UPDRS-III, *P* < 0.001; Modified H&Y, *P* = 0.028), with frailty being more prevalent in older patients with more severe PD. Frail PD patients showed significant cognitive impairment (*P* < 0.001). Although there was no significant statistical difference in medication dosage between the two groups (*P* = 0.076), a trend toward higher medication doses was observed in frail PD patients.

**TABLE 1:** Baseline characteristics of participants.

Characteristics	PD (*n* = 64)	Non-frailty (*n* = 34)	Frailty (*n* = 30)	*P* ^※^	HC (*n* = 17)	*P* ^※※^
Sex [female, *n* (%)]	30 (46.88)	16 (47.06)	14 (46.67)	0.975	11 (64.71)	0.191
Age(years, mean ± SD)	66.86 ± 6.97	64.29 ± 6.61	69.77 ± 6.17	**0.001**	62.88 ± 5.52	**0.034**
PD duration (years, median [IQR])	2 [2, 6]	2 [2, 4]	4 [1, 6]	0.418		
**Type, *n* (%)**						
Tremor (*n*, %)	6 (9.38)	5 (14.71)	1 (3.33)	0.297		
Tetanus (*n*, %)	14 (21.88)	7 (20.59)	7 (23.33)			
Mix (*n*, %)	44 (68.75)	22 (64.71)	22 (73.33)			
Modified H&Y (mean ± SD)	1.98 ± 0.62	1.82 ± 0.57	2.17 ± 0.64	**0.028**		
UPDRS III score (mean ± SD)	22.52 ± 7.88	18.74 ± 8.32	26.80 ± 4.51	**<0.001**		
LEDD (mg/day, mean ± SD)	441.66 ± 246.81	389.79 ± 196.89	500.45 ± 281.99	0.076		
Education (years, mean ± SD)	10.53 ± 3.91	10.71 ± 4.10	10.33 ± 3.68	0.709	11.41 ± 3.58	0.410
MOCA (mean ± SD)	17.53 ± 3.95	23.12 ± 2.71	18.30 ± 3.15	**<0.001**	22.18 ± 2.26	**<0.001**
**Fried’s frailty phenotype criteria**						
Weight loss, *n* (%)	19 (29.69%)	3 (8.82%)	16 (53.33%)	**<0.001**		
Weakness, *n* (%)	24 (37.50%)	2 (5.88%)	22 (73.33%)	**<0.001**		
Exhaustion, *n* (%)	26 (40.62%)	4 (11.76%)	22 (73.33%)	**<0.001**		
Slowness, *n* (%)	35 (54.69%)	10 (29.41%)	25 (83.33%)	**<0.001**		
Low activity, *n* (%)	27(42.19%)	11 (32.35%)	16 (53.33%)	0.149		

The Unified Parkinson’s Disease Rating Scale III (UPDRS III), the modified Hoehn and Yahr Staging Scale (Modified H&Y), levodopa equivalent daily dose (LEDD), Montreal Cognitive Assessment (MOCA), Health Controls (HC); *P*※-values for two-group comparison: Non-frailty vs. Frailty; *P*※※-values for two-group comparison: PD–HC. The bold values indicate statistical significance (*p* < 0.05).

**FIGURE 1:**
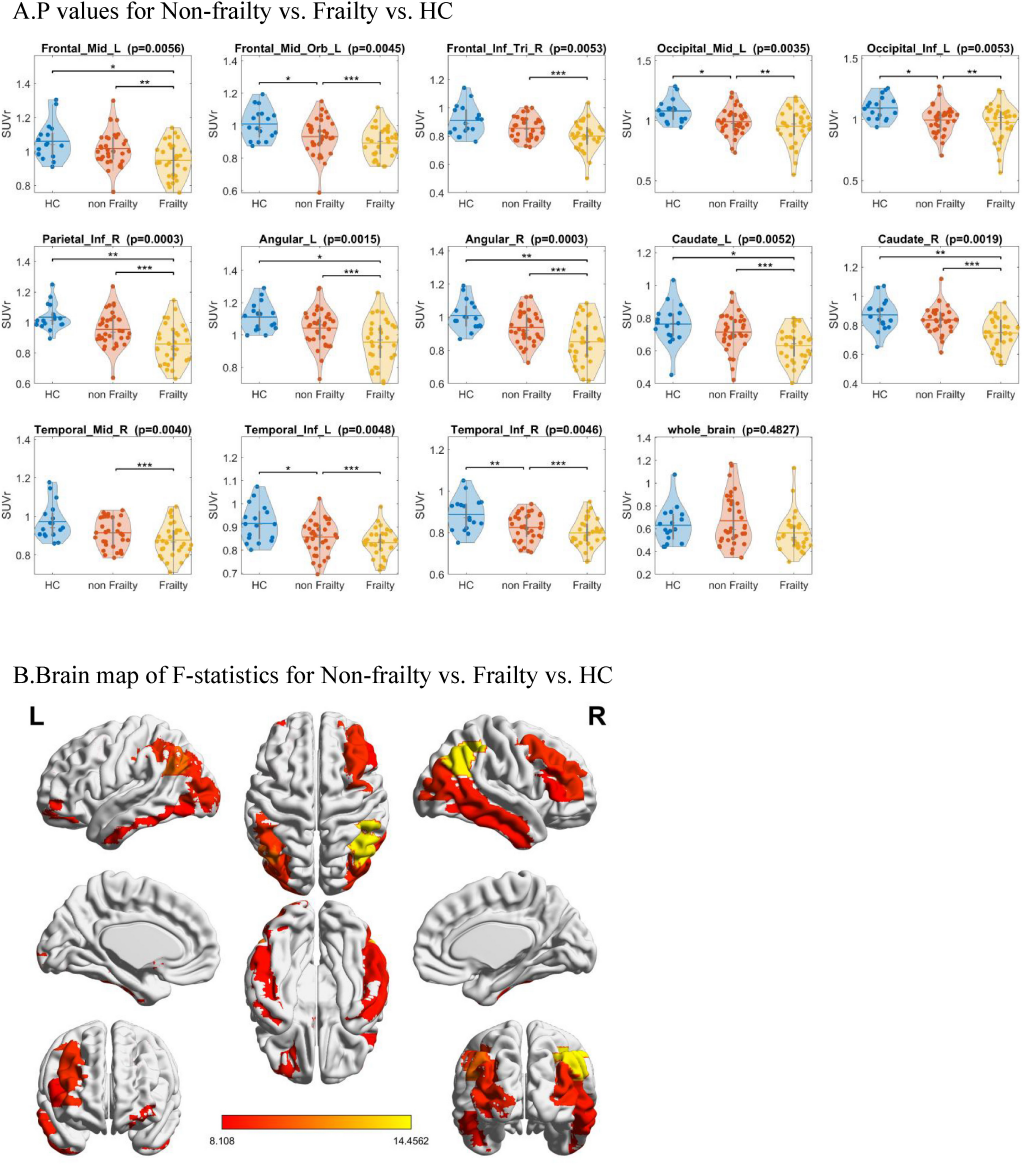
Chi-square analysis of the SUVr for three groups of participants. **(A)** *P*-values for Non-frailty vs. Frailty vs. HC. **(B)** Brain map of F-statistics for Non-frailty vs. Frailty vs. HC. SUVr, Standardized Uptake Value Ratio; HC, health controls. **(B)** From red to yellow, the difference in this brain area goes from small to large (FDR corrected *p* < 0.05). Reduced Glucose Metabolism in 13 Brain Regions of Frail PD vs. Non-Frail PD vs. HC: Frontal_Mid_L, Frontal_Mid_Orb_L, Frontal_Inf_Tri_R, Occipital_Mid_L, Occipital_Inf_L, Parietal_Inf_R, Angular_L, Angular_R, Caudate_L, Caudate_R, Temporal_Mid_R, Temporal_Inf_L, and Temporal_Inf_R.

As shown in Figures 1A,B, chi-square analysis was performed on the SUVr of the PD with frailty, non-frail PD, and healthy control groups. The results revealed that, compared to the non-frail PD group,13 brain regions in PD patients with frailty showed lower metabolism: Frontal_Mid_L (*P* = 0.0056),Frontal_Mid_Orb_L (*P* = 0.0045), Frontal_Inf_Tri_R (*P* = 0.0053), Occipital_Mid_L (*P* = 0.0035), Occipital_Inf_L (*P* = 0.0053), Parietal_Inf_R (*P* = 0.0003), Angular_L (*P* = 0.0015), Angular_R (*P* = 0.0003), Caudate_L (*P* = 0.0052), Caudate_R (*P* = 0.0019), Temporal_Mid_R (*P* = 0.0040), Temporal_Inf_L (*P* = 0.0048), and Temporal_Inf_R (*P* = 0.0046).

**FIGURE 2:**
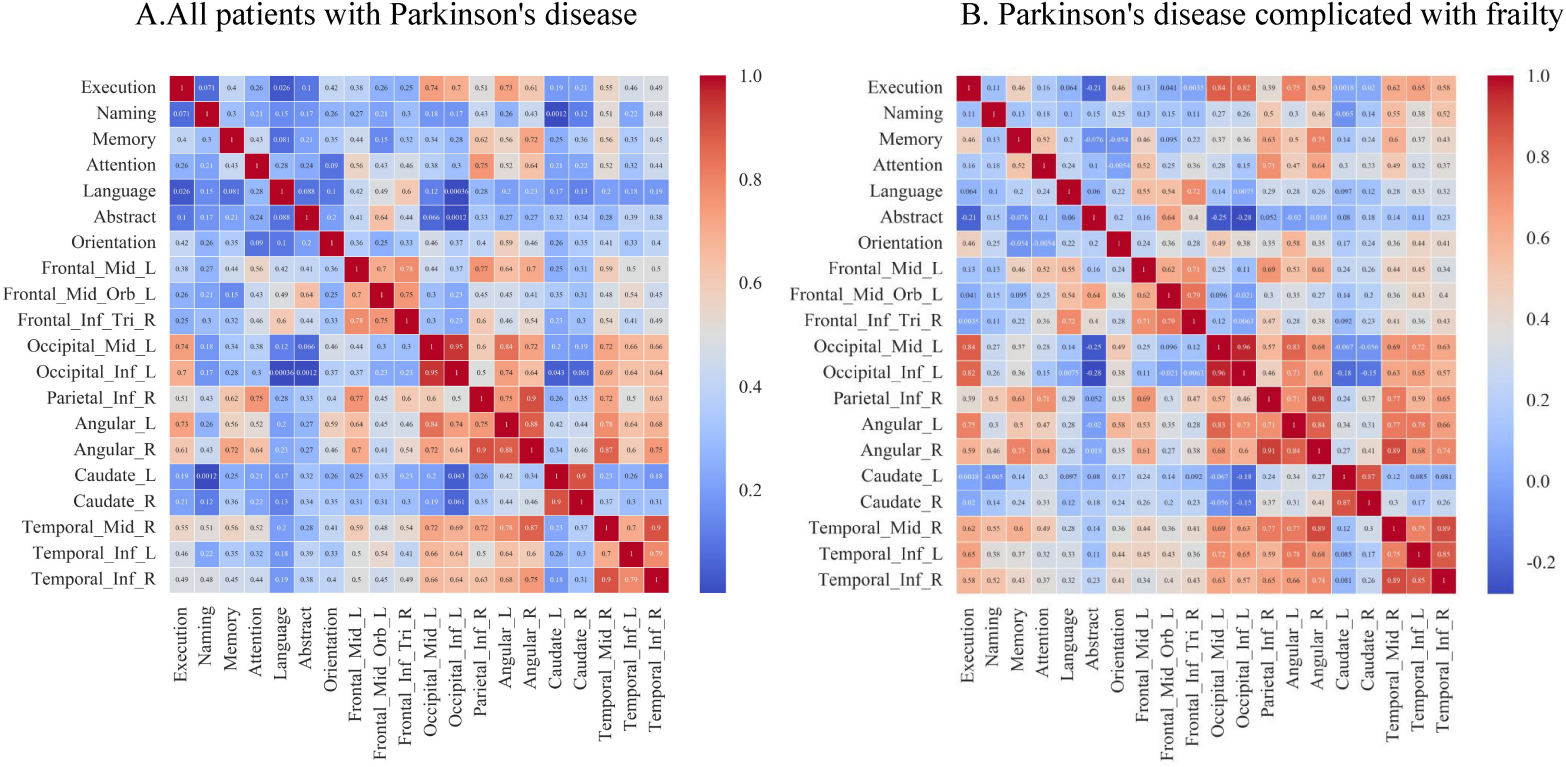
Pearson correlation analysis of the metabolic activity of ROIs and the seven cognitive domains. **(A)** All patients with Parkinson’s disease. **(B)** Parkinson’s disease complicated with frailty. The values of the correlation coefficients are presented in the form of a heatmap. The darker the color, the larger the value and the stronger the correlation.

Supplementary Tables 1, 2 and Figures 2A, B show Pearson correlation analyses between ROIs and the seven cognitive domains assessed in PD patients. In the entire PD cohort (*n* = 64),executive function was correlated with Occipital_Mid_L (*r* = 0.74), Occipital_Inf_L (*r* = 0.70), Parietal_Inf_R (*r* = 0.51), Angular_L (*r* = 0.73), Angular_R (*r* = 0.61), and Temporal_Mid_R (*r* = 0.55). Naming was correlated with Temporal_Mid_R (*r* = 0.51). Memory was associated with Parietal_Inf_R (*r* = 0.62), Angular_L (*r* = 0.56), Angular_R (*r* = 0.72), and Temporal_Mid_R (*r* = 0.56). Attention was correlated with Frontal_Mid_L (*r* = 0.56), Parietal_Inf_R (*r* = 0.75), Angular_L (*r* = 0.52), Angular_R (*r* = 0.64), and Temporal_Mid_R (*r* = 0.52). Language was associated with Frontal_Inf_Tri_R (*r* = 0.60). Abstraction was correlated with Frontal_Mid_Orb_L (*r* = 0.64). Orientation was associated with Angular_L (*r* = 0.59). In PD patients with frailty (*n* = 30), executive function was correlated with Occipital_Mid_L (*r* = 0.84), Occipital_Inf_L (*r* = 0.82), Angular_L (*r* = 0.75), Angular_R (*r* = 0.59), and Temporal_Mid_R (*r* = 0.62), and strengthening for Temporal_Inf_L (*r* = 0.65) and Temporal_Inf_R (*r* = 0.58). Naming was correlated with Temporal_Mid_R (*r* = 0.55) and enhanced by Temporal_Inf_R (*r* = 0.52). Memory remained correlated with Parietal_Inf_R (*r* = 0.63), Angular_L (*r* = 0.50), Angular_R (*r* = 0.75), and Temporal_Mid_R (*r* = 0.60). Attention remained associated with Frontal_Mid_L (*r* = 0.52), Parietal_Inf_R (*r* = 0.71), Angular_R (*r* = 0.64),while the correlations with Angular_L (*r* = 0.47) and Temporal_Mid_R (*r* = 0.49) weakened. Language was correlated with Frontal_Inf_Tri_R (*r* = 0.72), and associations with Frontal_Mid_L (*r* = 0.55) and Frontal_Mid_Orb_L (*r* = 0.54) were strengthened. Abstraction remained correlated with Frontal_Mid_Orb_L (*r* = 0.64),and orientation remained associated with Angular_L (*r* = 0.58).

Table 2 shows linear regression analysis of ROIs and UPDRS-III. In PD patients with frailty (*n* = 30),Temporal_Inf_R (*P* = 0.039) had a significant impact on UPDRS-III.

**TABLE 2:** Linear regression and VIF analysis of the regions of interests (ROIs) and Unified Parkinson’s Disease Rating Scale III (UPDRS III).

a. Linear regression analysis of the regions of interests (ROIs) and Unified Parkinson’s Disease Rating Scale III (UPDRS III).									
All patients/variables	Coefficient	Upper	Lower	*P*-value	Frail PD/variables	Coefficient	Upper	Lower	*P*-value
(Intercept)	43.095	7.518	78.673	0.021	(Intercept)	−17.815	−46.758	11.128	0.239
Frontal_Mid_Orb_L	6.496	−24.133	37.125	0.679	Frontal_Mid_Orb_L	21.642	−3.597	46.881	0.105
Parietal_Inf_R	−33.263	−65.828	−0.698	0.05	Caudate_L	−0.634	−21.934	20.665	0.954
Angular_L	−11.41	−46.406	23.585	0.525	Temporal_Inf_R	36.839	3.535	70.144	**0.039**
Caudate_L	−8.652	−35.943	18.64	0.537					
Temporal_Inf_R	27.458	−29.93	84.847	0.352					
Temporal_Inf_L	24.13	−8.68	56.95	0.16					
**b. VIF analysis of the regions of interests (ROIs) and Unified Parkinson’s Disease Rating Scale III (UPDRS III).**									
**All patients/variables**	**Tolerance**	**VIF**	**Frail PD/variables**		**Tolerance**	**VIF**			
Frontal_Mid_Orb_L	0.697	1.435	Frontal_Mid_Orb_L		0.829	1.207			
Parietal_Inf_R	0.402	2.489	Caudate_L		0.979	1.021			
Angular_L	0.314	3.187	Temporal_Inf_R		0.84	1.19			
Caudate_L	0.744	1.343							
Temporal_Inf_R	0.467	2.141							

VIF, variance inflation factor; Tolerance = 1/VIF. A VIF value > 10 or Tolerance < 0.1 indicates severe multicollinearity; a VIF value between 5 and 10 indicates moderate multicollinearity. The bold values indicate statistical significance (*p* < 0.05).

## 4 Discussion

Frailty and Parkinson’s disease (PD) share overlapping pathophysiological mechanisms. Frailty is associated with dysfunction of the central nervous system, sympathetic nervous system, and endocrine system, as well as imbalances in homeostasis and energy metabolism (Walston et al., 2006). Neuroimmune and inflammatory changes play important roles in the onset and progression of both conditions. Studies have shown that certain cytokines and leukocyte subtypes are associated with the development of frailty (Vatic et al., 2020). In our study, patients with PD and frailty exhibited more severe cognitive impairment. Furthermore, brain metabolic changes associated with frailty may aggravate PD-related motor symptoms. To our knowledge, this is the first report of FDG-PET/MR-defined metabolic patterns in PD patients with frailty. While the underlying neuropathological mechanisms remain unclear, integrated PET/MR imaging offers a powerful tool for identifying relevant regions of interest.

This study explored the brain metabolic patterns of PD patients with frailty using FDG-PET/MR imaging and identified significant hypometabolism in 13 brain regions: the left middle frontal gyrus (Frontal_Mid_L), left medial orbital frontal gyrus (Frontal_Mid_Orb_L), right inferior frontal gyrus, triangular part (Frontal_Inf_Tri_R), left middle occipital gyrus (Occipital_Mid_L), left inferior occipital gyrus (Occipital_Inf_L), right inferior parietal lobule (Parietal_Inf_R), left and right angular gyri (Angular_L and Angular_R), left and right caudate nuclei (Caudate_L and Caudate_R), right middle temporal gyrus (Temporal_Mid_R), and left and right inferior temporal gyri (Temporal_Inf_L and Temporal_Inf_R). Notably, nearly all of these regions are not adjacent to one another.

Pearson correlation analyses were conducted between the ROIs and the seven cognitive dimensions. In frail PD patients, executive function was associated with Occipital_Mid_L, Occipital_Inf_L, Angular_L, Angular_R,and Temporal_Mid_R. Additionally, associations with Parietal_Inf_R weakened, while those with Temporal_Inf_L and Temporal_Inf_R strengthened. Naming was related to Temporal_Mid_R and showed enhanced association with Temporal_Inf_R. Memory remained associated with Parietal_Inf_R,Angular_L, Angular_R, and Temporal_Mid_R. Attention was associated with Frontal_Mid_L, Parietal_Inf_R, and Angular_R, but showed reduced correlation with Angular_L and Temporal_Mid_R. Language was associated with Frontal_Inf_Tri_R and showed stronger connections with Frontal_Mid_L and Frontal_Mid_Orb_L. Abstract reasoning remained associated with Frontal_Mid_Orb_L, and orientation with Angular_L.

The pathway linking visual stimulation from the occipital to temporal lobe and subsequently to the limbic system and caudate nucleus plays a critical role in executive dysfunction (Grill et al., 2021; Nishimura et al., 2022), which is prominent in both PD and frailty (Muslimovic et al., 2005). Temporal_Mid_R and Temporal_Inf_R are essential for semantic and auditory processing (Gasca-Salas et al., 2019), and damage to these areas impairs naming ability (Carvalho de Abreu et al., 2023). Parietal_Inf_R is involved in memory and attention networks. Disruptions in frontal-parietal synchronization impair working memory (Vatansever et al., 2017; Rubinstein et al., 2021), and parietal lesions can lead to attentional deficits (Fiebelkorn and Kastner, 2020).

Memory was related to Temporal_Mid_R and bilateral angular gyrus. The temporal lobe is related to cognitive function. The hippocampus is located in the medial temporal lobe and is associated with other brain regions in the temporal lobe (Cowan et al., 2020). The angular gyrus supports reading,semantic memory,spatial cognition,and memory retrieval (Matchin et al., 2019). As a hub in the default mode network (DMN), it integrates multimodal contextual details vital for episodic memory (Ramanan et al., 2018; Benoit and Schacter, 2015). The interaction between the angular gyrus and the hippocampus may generate rich memory representations, and the connection between the angular gyrus and the hippocampal formation is the most important (Coughlan et al., 2023), possibly interacting with the hippocampus via reciprocal connections (Thakral et al., 2020; Wang et al., 2014). This may explain its central role in frailty-related memory impairment. Amyloid-β deposition in the angular gyrus and occipital cortex also contributes to cognitive decline (Stevens et al., 2022).

Frontal_Mid_L is associated with attention and is responsive to emotional stimuli (Schupp et al., 2003). Prefrontal delta and theta activity is linked to cognitive function in PD (Cavanagh and Frank, 2014; Zavala et al., 2018; Senftleben and Scherbaum, 2021). The spectral analysis of frontal electroencephalogram can predict the cognitive dysfunction of PD (Singh et al., 2021). The prefrontal stimulation improves cognition in PD animal models (Kelley et al., 2018).

Frontal_Inf_Tri_R is involved in semantic processing (Deng et al., 2016). Frailty and PD share frontal lobe vulnerabilities affecting language and abstraction. However, frailty may more strongly impact executive and attentional domains, while orientation remains comparable between groups. These domain-specific effects may reflect a neural compensation process in the transition from non-frailty to frailty, mediated by neuroplasticity (Sun et al., 2016).

The cholinergic system, including basal forebrain projections and striatal interneurons, is critical for cognition (Ballinger et al., 2016; Zhang et al., 2016; Power et al., 2024). The posterior cortex plays a central role in cholinergic circuits (Hilker et al., 2005; van der Zee et al., 2022). The observed hypometabolism in posterior cortical regions may reflect cholinergic denervation, which we hypothesize contributes to frailty in PD.

Given the importance of the UPDRS-III in PD evaluation, we examined its associations with ROIs. Linear regression revealed a significant association between UPDRS-III scores and Temporal_Inf_R in frail PD patients. Temporal_Inf_R plays a fundamental role in visual cognition. Importantly, its function in visuo-motor cross-modal integration represents a core mechanism associated with motor dysfunction in PD (Genovese et al., 2025). Alterations in large-scale brain network efficiency in PD patients are closely correlated with post-medication UPDRS-III scores. As a key node in visuo-motor network integration, Temporal_Inf_R demonstrates robust functional connectivity with the basal ganglia, frontal motor cortex, and other motor-related regions. The functional state of Temporal_Inf_R modulates the motor regulatory capacity of the global brain network, thereby correlating with motor symptom severity as measured by the UPDRS-III (Li et al., 2019).

It is well-established that the amplitude of low-frequency fluctuations (ALFF) is widely used to assess spontaneous neural activity under both physiological and pathological conditions (Wang et al., 2020). Furthermore, compared with healthy controls, PD patients exhibit increased ALFF in bilateral motor areas—regions primarily evaluated by the UPDRS-III (Yang et al., 2023). Previous studies have reported elevated ALFF values in regions including Temporal_Inf_R in PD patients (Wang et al., 2020). As a key metric of spontaneous neural activity, the extent of ALFF abnormalities is closely associated with the degree of motor impairment in PD. Together, these findings support the association between functional abnormalities in Temporal_Inf_R and motor symptoms assessed by the UPDRS-III, underscoring its pivotal role in the development and progression of frailty in PD patients.

Furthermore, while neuroanatomical and functional damage is typically irreversible, as is the case in the chronic neurodegenerative course of PD (Zhang et al., 2005; Kalia and Lang, 2015), increasing evidence indicates that frailty is a dynamic condition that can transition into a non-frail state (Gill et al., 2006; Racey et al., 2021). This suggests the possibility of prevention or treatment. Exercise interventions have been shown to improve mobility and physical function in frail older adults, with high-intensity physical activity yielding greater benefits than low-intensity exercise (Theou et al., 2011). Some researchers have even proposed that exercise may be more beneficial than any other clinical intervention (de Vries et al., 2012). Nutritional support and management of depressive symptoms also serve as effective strategies to mitigate frailty. Clinicians should enhance awareness and assessment of frailty to better implement preventive and therapeutic strategies (Fairhall et al., 2011). Multidomain interventions in frail adults have shown that taking multivitamin and multimineral supplements for over 6 months can reverse physical frailty (Ng et al., 2015). The Mediterranean diet, rich in antioxidants, may reduce oxidative stress—a key factor in muscle atrophy and fiber loss—thus offering another potential avenue for frailty management.

Given the high prevalence of frailty among individuals with PD (Ahmed et al., 2008; Roland et al., 2012; Liotta et al., 2017; Renne and Gobbens, 2018), and its adverse effects on prognosis (Fried et al., 2001; Rothman et al., 2008; Makizako et al., 2015), targeted intervention for frailty in PD is warranted. Our study identified the brain metabolic patterns associated with frailty in PD, with a particular focus on their relationships with cognitive and motor function. We also discussed the relevant neuropathophysiological mechanisms, offering strong evidence to guide future animal and clinical studies, and potential targets for frailty interventions. By investigating the neurophysiological basis of frailty in PD through brain metabolic imaging, our goal is to provide neuroimaging evidence for precision interventions.

## 5 Conclusion

This study is the first to demonstrate that patients with Parkinson’s disease and frailty exhibit hypometabolism in multiple brain regions, particularly the frontal, parietal, temporal cortices, and caudate nucleus. These metabolic changes are significantly associated with cognitive impairment, and motor function scores, suggesting that these regions may be involved in the neuropathological processes of frailty in PD. These findings may provide potential neuroimaging biomarkers and therapeutic targets for intervention strategies.

## 6 Limitations

This study has several limitations that should be acknowledged. First, frailty is often accompanied by multiple comorbid factors in clinical practice, and these factors are not mutually exclusive. Given the potential bidirectional interactions between frailty and PD, although frailty assessments were performed during the “on” state of PD (when dopaminergic medications are effective), the reciprocal influence between the two conditions cannot be fully ruled out. Second, the age distribution differed between the PD patient group and healthy control group, which may have introduced confounding effects that warrant consideration. Additionally, the relatively limited sample size may compromise the statistical power for detecting significant differences in secondary outcomes. Finally, all participants were recruited from a single center, which may restrict the external validity and generalizability of the findings to the broader PD population. To further elucidate the underlying mechanisms and definitively establish causality, future studies should employ a rigorous multi-method approach. This includes employing animal models and longitudinal follow-up designs to clarify the pathophysiological links between Parkinson’s disease (PD) and frailty. In parallel, longitudinal cohort studies are needed to track cerebral metabolic changes before the onset of frailty, along with interventional studies that examine whether improvements in frailty—resulting from interventions such as rehabilitation training—are associated with the recovery of cerebral metabolism. We anticipate that subsequent research will address these limitations and build upon the findings of the present study.

## Data Availability

The original contributions presented in this study are included in this article/Supplementary material, further inquiries can be directed to the corresponding authors.

## Footnotes

1
http://www.fil.ion.ucl.ac.uk/spm/software/spm8
