# Supplementary material for: 18F-FDG PET/MR reveals specific brain metabolic features in Parkinson’s disease with frailty
Source: Front Aging Neurosci. 2026 Jan 22;17:1624203. doi: 10.3389/fnagi.2025.1624203 (PMC12872807; doi:10.3389/fnagi.2025.1624203)
Supplement: Supplementary file 1 [file Data_Sheet_1.docx]

Supplementary Table 1.Pearson correlation analysis of the metabolic activity of ROIs and the seven cognitive domains in all patients with Parkinson's disease

| Domains | Frontal_Mid_L | Frontal_Mid_Orb_L | Frontal_Inf_Tri_R | Occipital_Mid_L | Occipital_Inf_L | Parietal_Inf_R | Angular_L | Angular_R | Caudate_L | Caudate_R | Temporal_Mid_R | Temporal_Inf_L | Temporal_Inf_R |
| --- | --- | --- | --- | --- | --- | --- | --- | --- | --- | --- | --- | --- | --- |
| Execution | 0.38 | 0.26 | 0.25 | 0.74 | 0.70 | 0.51 | 0.73 | 0.61 | 0.19 | 0.21 | 0.55 | 0.46 | 0.49 |
| Naming | 0.27 | 0.21 | 0.3 | 0.18 | 0.17 | 0.43 | 0.26 | 0.43 | 0.0012 | 0.12 | 0.51 | 0.22 | 0.48 |
| Memory | 0.44 | 0.15 | 0.32 | 0.34 | 0.28 | 0.62 | 0.56 | 0.72 | 0.25 | 0.36 | 0.56 | 0.35 | 0.45 |
| Attention | 0.56 | 0.43 | 0.46 | 0.38 | 0.30 | 0.75 | 0.52 | 0.64 | 0.21 | 0.22 | 0.52 | 0.32 | 0.44 |
| Language | 0.42 | 0.49 | 0.60 | 0.12 | 0.00036 | 0.28 | 0.20 | 0.23 | 0.17 | 0.13 | 0.20 | 0.18 | 0.19 |
| Abstract | 0.41 | 0.64 | 0.44 | 0.07 | 0.0012 | 0.33 | 0.27 | 0.27 | 0.32 | 0.34 | 0.28 | 0.39 | 0.38 |
| Orientation | 0.36 | 0.25 | 0.33 | 0.46 | 0.37 | 0.40 | 0.59 | 0.46 | 0.26 | 0.35 | 0.41 | 0.33 | 0.40 |

A Pearson coefficient greater than 0.5 indicates that there is a relatively high correlation between the variables.

Supplementary Table 2.Pearson correlation analysis of the metabolic activity of ROIs and the seven cognitive domains in Parkinson's disease complicated with frailty

| Dimensions | Frontal_Mid_L | Frontal_Mid_Orb_L | Frontal_Inf_Tri_R | Occipital_Mid_L | Occipital_Inf_L | Parietal_Inf_R | Angular_L | Angular_R | Caudate_L | Caudate_R | Temporal_Mid_R | Temporal_Inf_L | Temporal_Inf_R |
| --- | --- | --- | --- | --- | --- | --- | --- | --- | --- | --- | --- | --- | --- |
| Execution | 0.13 | 0.04 | 0.0035 | 0.84 | 0.82 | 0.39 | 0.75 | 0.59 | 0.0018 | 0.02 | 0.62 | 0.65 | 0.58 |
| Naming | 0.13 | 0.15 | 0.11 | 0.27 | 0.26 | 0.50 | 0.30 | 0.46 | -0.06 | 0.14 | 0.55 | 0.38 | 0.52 |
| Memory | 0.46 | 0.09 | 0.22 | 0.37 | 0.36 | 0.63 | 0.50 | 0.75 | 0.14 | 0.24 | 0.60 | 0.37 | 0.43 |
| Attention | 0.52 | 0.25 | 0.36 | 0.28 | 0.15 | 0.71 | 0.47 | 0.64 | 0.30 | 0.33 | 0.49 | 0.32 | 0.37 |
| Language | 0.55 | 0.54 | 0.72 | 0.14 | 0.01 | 0.29 | 0.28 | 0.26 | 0.10 | 0.12 | 0.28 | 0.33 | 0.32 |
| Abstract | 0.16 | 0.64 | 0.4 | -0.25 | -0.28 | 0.05 | -0.02 | 0.02 | 0.08 | 0.18 | 0.14 | 0.11 | 0.23 |
| Orientation | 0.24 | 0.36 | 0.28 | 0.49 | 0.38 | 0.35 | 0.58 | 0.35 | 0.17 | 0.24 | 0.36 | 0.44 | 0.41 |

A Pearson coefficient greater than 0.5 indicates that there is a relatively high correlation between the variables.
